# Revisiting Diagnostic Criteria for Bilateral Vestibulopathy: A New Comprehensive Instrumental Model

**DOI:** 10.3390/audiolres14060082

**Published:** 2024-11-16

**Authors:** Leonardo Manzari, Nicola Ferri, Marco Tramontano

**Affiliations:** 1 MSA ENT Academy Center, 03043 Cassino, Italy; 2 Department of Biomedical and Neuromotor Sciences (DIBINEM), Alma Mater University of Bologna, 40138 Bologna, Italy; nicola.ferri11@unibo.it (N.F.); marco.tramontano@unibo.it (M.T.); 3 Unit of Occupational Medicine, IRCCS Azienda Ospedaliero-Universitaria di Bologna, 40138 Bologna, Italy

**Keywords:** bilateral vestibulopathy, head impulse test, evoked potentials, vestibular system, diagnosis, dizziness

## Abstract

**Background:** Bilateral vestibulopathy (BVP) is a disabling condition characterized by a deficit in vestibular function on both sides. Current diagnostic criteria consider instrumental data only from horizontal canals, excluding vertical canals and otolithic function, with the possibility of not including some variants of BVP. This study aims to evaluate vestibular functions in people with chronic vestibular syndrome through a comprehensive battery of tests. **Methods:** This diagnostic accuracy study included patients who met criteria for probable BVP. The index test included a thorough evaluation of the vestibular system, using the video Head Impulse Test (vHIT) to measure the gain of the angular vestibulo-ocular reflex (aVOR) in all six semicircular canals and the cervical and ocular vestibular-evoked myogenic potentials (VEMPs) to assess otolith function. The diagnostic criteria established by the Barany Society were considered the standard reference, including only the horizontal vHIT as an instrumental assessment. **Results:** 78 patients (41 male, age 61.40 ± 12.99) were enrolled. The Barany criteria showed a low ability to rule out BPV (sensitivity = 46%). The median Dizziness Handicap Inventory (DHI) varied from 66 to 69 among the models studied, and a significant difference in DHI scores between positive and negative tests was observed for the Barany criteria and the six-canals vHIT model. **Conclusions:** Our findings highlight the potential to transform BPV diagnostic criteria. The identification of new bilateral vestibular dysfunction variants through improved diagnostic tools calls for revising current criteria, with promising implications for patient care and understanding of etiological and prognostic aspects.

## 1. Introduction

Bilateral vestibulopathy (BVP), also referred to as Bilateral Vestibular Hypofunction (BVH), is a clinical condition characterized by a significant reduction or complete loss of vestibular function on both sides [1]. This disorder affects the vestibular receptors or the neural pathways responsible for transmitting vestibular information to the central nervous system, leading to symptoms such as chronic dizziness, imbalance, oscillopsia, and, in some cases, a profound sense of cognitive fog or mental cloudiness [2,3,4]. The term “fog” is often used by patients to describe the sensation of disorientation and mental sluggishness accompanying BVP’s physical symptoms [5,6].

Before the development of new instrumental tests and the Barany criteria, the prevalence of BVP in the adult population of the United States was estimated to be approximately 28 cases per 100,000 people, [7] with the incidence ranging between 4% and 7%. The age range of individuals affected by BVP spans from young adults to the elderly, with the underlying etiology playing a significant role in determining the onset of symptoms. For cases of acquired BVP [8], the average age at diagnosis is typically between 50 and 60 years [9].

Indeed, etiologies of BVP are diverse, encompassing a wide range of conditions, including toxic, infectious, traumatic, bilateral otologic diseases, and congenital syndromes [1]. Given this broad spectrum of potential causes, diagnosing BVP can be particularly challenging, especially in its early stages where symptoms may be subtle or attributed to other conditions. BVP is associated with significant morbidity, affecting patients’ ability to perform daily activities and diminishing their overall quality of life [10]. The most common symptoms reported by patients include chronic postural imbalance, which worsens in low-visibility conditions or when walking on uneven surfaces, and oscillopsia, a visual disturbance where objects appear to oscillate or move when the head is in motion. Oscillopsia occurs due to the impaired vestibulo-ocular reflex (VOR), which, in normal conditions, stabilizes images on the retina during head movements.

The diagnosis of BVP typically relies on clinical assessment and a combination of vestibular function tests. According to the Bárány Society’s 2017 guidelines [1], the diagnosis of BVP is based on chronic clinical symptoms and bilateral reduced or absent angular VOR (aVOR) function. The altered function of aVOR can be documented using a video Head Impulse Test (vHIT), caloric response, or rotatory chair. Additionally, the diagnosis requires the exclusion of other potential causes of these symptoms. It is important to note that the Bárány criteria consider only the VOR function of the horizontal semicircular canals and not the vertical semicircular canals or other instrumental tests for additional vestibular functions.

We hypothesize that the current diagnostic criteria may not fully capture the heterogeneity of BVP, as evidenced by patients who present functional VOR gain and significant vestibular dysfunction as detected by other tests, such as cervical or ocular vestibular-evoked myogenic potentials (VEMPs). Indeed, Fujimoto and colleagues had already described a bilateral absence of VEMPs in the presence of normal caloric response [11]. Furthermore, aVOR dysfunction could also affect vertical semicircular canals (SCC) [12].

This study aimed to evaluate aVOR and otolithic functions in people suffering from chronic vestibular syndrome and perceived severe dizziness-related handicap through a comprehensive battery of instrumental tests.

## 2. Materials and Methods

### 2.1. Study Design

This diagnostic accuracy study included the medical records of patients who met the diagnostic criteria for probable bilateral vestibulopathy. Medical records were reviewed at the MSA ENT Academy Center, a tertiary referral center specializing in vestibular disorders, from July 2022 to December 2023. Data collection was planned after the index test and reference standard were performed. This study was approved by the MSA Institutional Review Board, and it adheres to the guidelines established by the Standards for Reporting Diagnostic Accuracy Studies (STARD) [13].

### 2.2. Participants

Participants formed a consecutive series obtained from MSA ENT Academy Center internal records. Inclusion criteria required patients to exhibit symptoms such as unsteadiness while walking or standing, movement-induced blurred vision, absence of symptoms while sitting or lying under static conditions, and a Dizziness Handicap Inventory [14] score > 54.

### 2.3. Index Test

The index test consisted of a comprehensive vestibular assessment, including the vHIT, to measure aVOR gain across all six SSC and cervical and ocular VEMPs to assess otolith function. The Barany Society diagnostic criteria represent the reference standard, using only horizontal vHIT as instrumental assessment.

### 2.4. Video Head Impulse Test

The vHIT (ICS Impulse, Otometrics/Natus, Taastrup, Denmark) measured the aVOR gain across the six semicircular canals (SCC). The evaluation process adhered to strict protocols to ensure data consistency and accuracy [15]. An expert clinician (LM) conducted the tests.

During the aVOR evaluation, patients were instructed to focus on a fixed point on the wall 1 m away. Room lighting was adjusted to prevent pupil image distortion due to reflections. Approximately 14 unpredictable, brief horizontal head impulses were applied to each side, with rotations of 10–15 degrees and peak velocities between 140 and 220 deg/s. Eye and head velocities were recorded, with the gain (eye velocity over head velocity at peak acceleration) as the primary measure, and no compensatory saccades were expected under normal conditions. For vertical SCC evaluation, the head was positioned 35 degrees left for Right Anterior-Left Posterior (RALP) and 35 degrees right for Left Anterior-Right Posterior (LARP) testing. Head movements were quick and minimal, with care to avoid goggle slippage. We considered functional aVOR when the gain was ≥0.60.

### 2.5. Vestibular-Evoked Myogenic Potentials (VEMPs)

The assessment of otolith function was performed using ocular and cervical VEMPs. Specifically, for evaluating the utricular macula, the focus was on the n10 component, characterized as a negative (excitatory) potential with an amplitude ranging from 5 to 10 μV. This response is considered crossed, reflecting the activity of the inferior oblique eye muscles, and is recorded using surface electromyography electrodes placed on the skin beneath the eyes. The stimuli used to evoke oVEMPs included bone-conducted vibration (BCV), applied to the midline of the forehead at the hairline (Fz), and air-conducted sound (ACS).

Based on anatomical and physiological studies, BCV at 500 Hz and ACS have been shown to preferentially activate irregular otolithic afferent neurons, particularly those associated with the utricle. Furthermore, evidence supports the involvement of utricular-ocular projections in generating these responses. As such, the n10 component of the oVEMP is considered a reliable indicator of utricular function in response to these stimuli.

To evaluate the symmetry of the responses between the left and right sides, the asymmetry ratio (AR) was calculated. This AR was determined using a modified version of the standard Jongkees formula, commonly employed in vestibular testing. The formula for AR is as follows: AR = 100 × (larger VEMP+smaller VEMP)/ (larger VEMP − smaller VEMP).

This calculation was applied to the oVEMP n10 amplitude and the cVEMP p13–n23 amplitude, providing a quantitative measure of vestibular asymmetry. The bilateral absence of n10 to oVEMPs and p13–n25 to cVEMPs also points to BVP and determines an asymmetry ratio value of 0.

### 2.6. Reference Standard (Barany Diagnostic Criteria for BPV) [1]

- A. Chronic vestibular syndrome with the following symptoms:
  1. Unsteadiness when walking or standing plus at least one of 2 or 3;
  2. Movement-induced blurred vision or oscillopsia during walking or quick head/body movements and/or;
  3. Worsening of unsteadiness in darkness and/or on uneven ground.
  B. No symptoms while sitting or lying down under static conditions.
  C. Bilaterally reduced or absent aVOR function documented by:
- Bilaterally pathological horizontal aVOR gain <0.6, measured by the vHIT or scleral-coil technique and/or;
- Reduced caloric response (sum of bithermal max. peak SPV on each side <6°/s) and/or;
- Reduced horizontal aVOR gain <0.1 upon sinusoidal stimulation on a rotatory chair (0.1 Hz, Vmax = 50°/s) and a phase lead >68 degrees (time constant <5 s).
  D. Not better accounted for by another disease.

### 2.7. Statistical Analysis

Diagnostic accuracy was evaluated by comparing the sensitivity, specificity, and ROC area for the index test model (which includes both VOR and VEMPs testing) against the Bárány criteria model (based on horizontal vHIT only). Missing data were handled by excluding incomplete records from the final analysis.

We also investigated a reverse diagnostic test accuracy scenario, considering the new comprehensive model an extension of the Barany criteria and, thus, as the new reference standard for this research purpose

## 3. Results

After screening 1169 medical records, 78 patients (41 male, mean age 61.40 ± 12.99) who met the diagnostic criteria for probable BVP were included in the analysis. Clinical and demographic information are reported in Table 1.

The diagnostic classification of BPV according to the proposed new criteria against the Barany criteria is reported in Table 2.

Table 3 reports the diagnostic accuracy properties of each of the three proposed criteria against the Barany criteria. Since true negative and false positive are structural zeros, no models could be created to fit the comprehensive criteria data (i.e., the combination of three index tests).

### 3.1. Comprehensive Model—Gold Standard Scenario

In this alternative scenario, where the union of the models analyzed in Table 3 has been considered the reference standard, the Barany criteria would have a high rate of false negatives (sensitivity = 46%), resulting in a low ability to rule out BPV (Table 4).

### 3.2. Clinical Outcomes

**Figure 1:**
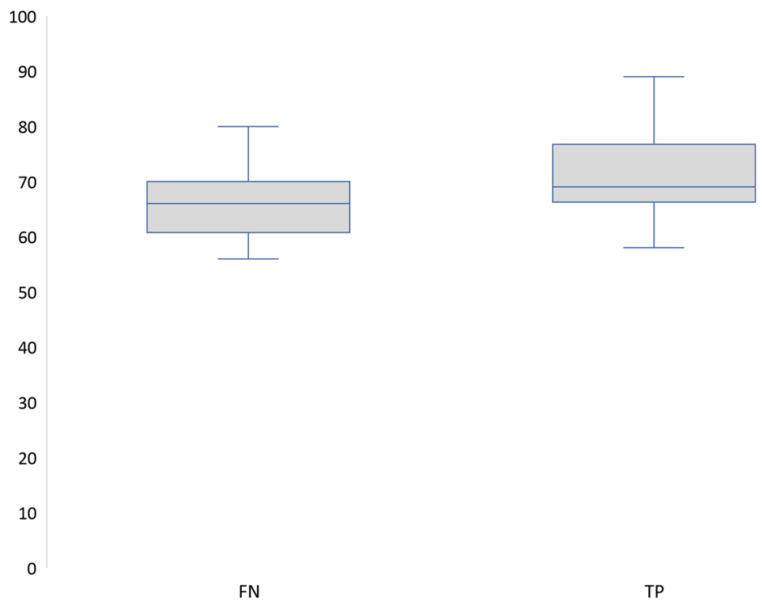
DHI median scores between false negatives and true positives (Barany criteria).FN: False negatives, TP: True positives.

According to the Barany criteria, the median DHI scores of the true positive group (*n* = 36) and the false negative group (*n* = 42) were significantly different (Figure 1) as analyzed with the Wilcoxon rank-sum test (z = −2.508, exact *p* = 0.0117).

**Figure 2:**
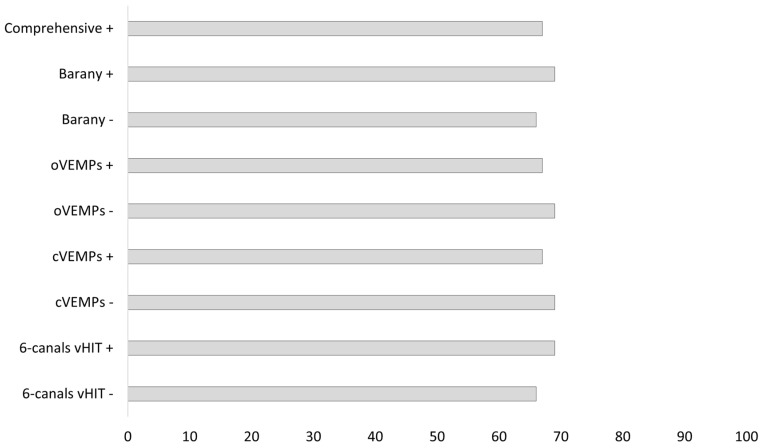
DHI score across different criteria.

Figure 2 reports the DHI median score for each of the models considered.

**Figure 3:**
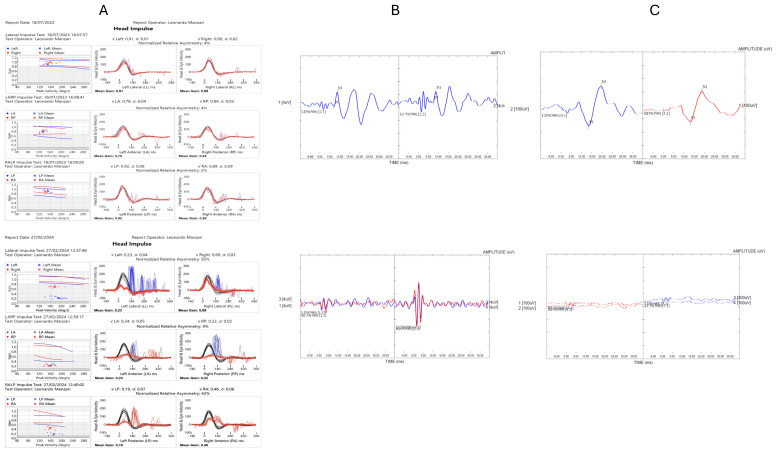
Comparison between normal and abnormal results. The left upper part (**A**) shows the normal results of vHIT, with the lower part displaying the dysfunctional results. The central upper part (**B**) presents the normal cVEMPs results, while the lower part shows the abnormal bilateral response. The right upper part (**C**) displays the normal oVEMPs results, with the lower part showing the abnormal bilateral response.

Median DHI scores ranged between 66 and 69 across the models analyzed. The difference in DHI between positive and negative tests was significant only for the Barany criteria (z = −2.508, *p* = 0.0117) and the six-canals vHIT model (z = −2.376, *p* = 0.0167). Figure 3 presents a comparison between normal and abnormal results.

**Figure 4:**
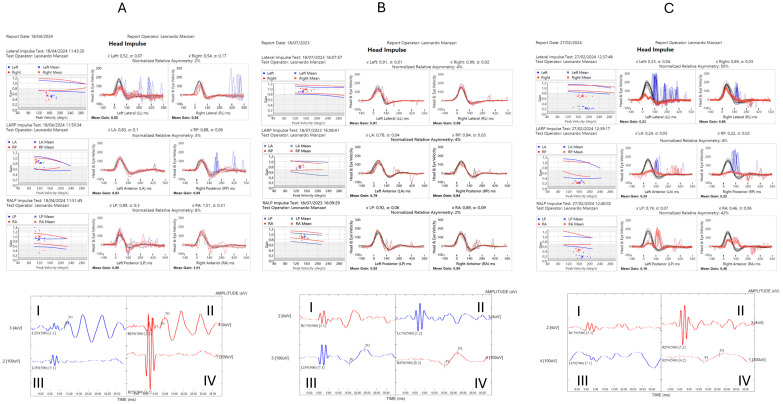
Possible combinations of the comprehensive model assessment in patients with bilateral vestibulopathy.

Instead, Figure 4 presents the possible combinations of the comprehensive model assessment.

The left upper part (**A**) shows the results of vHIT testing of both left and right semicircular canals: eye velocity (red traces) and head velocity (black traces) versus time. The signs of head velocity and of eye velocity for right and leftward impulses, LARP and RALP impulses, have been inverted for easier comparison. The patient shows abnormal responses for head impulses in the plane of the left horizontal semicircular canal clear reduction of the slow phase velocity of the VOR followed by corrective saccades. The patient in contrast shows normal responses for head impulses to right sides and in the plane of the left vertical semicircular canals—eye velocity matched head velocity closely. The left bottom part (**A**) shows oVEMPs (I and II) and cVEMPs (III and IV) to bone-conducted vibration: the time of the n10 response shows symmetrical amplitude beneath the eyes of the patient, indicating normal utricular macula function at the striola. The lowest traces (III and IV) show the cVEMPs to 500 Hz Fz BCV where the response time for the p13 and n23 complex should be expected. These responses are absent in this subject, indicating absent saccular macula function at the striola. The central upper part (**B**) shows the results of vHIT testing of both left and right semicircular canals: eye velocity (red traces) and head velocity (black traces) versus time. The signs of head velocity and of eye velocity for right and leftward impulses, LARP and RALP impulses, have been inverted for easier comparison. The patient shows normal responses for head impulses to both sides—eye velocity matched head velocity closely. The central bottom part (**B**) shows oVEMPs (I and II) and cVEMPs (III and IV) to bone-conducted vibration where the response time of the n10 response should be expected, which shows absent negative wave beneath the eyes of the patient, indicating absent utricular macula function at the striola. The bottom rows (III and IV) show the cVEMPs to 500 Hz Fz BCV: the time of the p13 and n23 responses are marked with small vertical lines. These responses are normal in this individual. The right upper part (**C**) shows the results of vHIT testing of both left and right semicircular canals: eye velocity (red traces) and head velocity (black traces) versus time. The signs of head velocity and of eye velocity for right and leftward impulses, LARP and RALP impulses, have been inverted for easier comparison. This patient shows clear abnormal responses for head impulses in the plane of the six semicircular canals with a clear reduction of the slow phase velocity of the VOR followed by corrective saccades. The right bottom—part (**C**) shows oVEMPs (I and II) and cVEMPs (III and IV) to bone-conducted vibration, where the response time of the n10 response should be expected, but shows an absent negative wave beneath the eyes of the patient, indicating absent utricular macula function at the striola. The lowest rows (III and IV) show the cVEMPs to 500 Hz Fz BCV, where the response time of the p13 and n23 responses should be expected. This part of both traces reveals absent positive–negative waves over the sternocleidomastoid muscle of the patient, indicating absent saccular macula function at the striola.

## 4. Discussion

This study aimed to evaluate semicircular and otolithic functions using a comprehensive battery of tests in people suffering from BVP. VOR gain is a valuable indicator of semicircular canal function. However, it may not fully capture the spectrum of vestibular dysfunction present in patients with BVP. The detection of significant otolithic abnormalities in a subset of patients with normal VOR gain suggests that otolith function testing should be considered an essential component of the diagnostic process. Given the variability in vestibular function observed among patients with BVP, there is a compelling case for revising the current diagnostic criteria to include a broader range of vestibular tests. Including VEMP testing, in particular, could help identify patients with BVP subtypes that might otherwise go undiagnosed. Such a revision would enhance diagnostic accuracy and facilitate earlier intervention and more targeted rehabilitation strategies. Understanding the full spectrum of vestibular deficits in BVP is crucial for developing effective treatment plans. Patients with otolithic dysfunction, for example, may benefit from different rehabilitation approaches compared to those with primary semicircular canal dysfunction. Thus, tailoring treatment to the specific vestibular deficits identified in each patient could lead to better outcomes and improved quality of life.

This study opens several avenues for future research. Longitudinal studies are needed to determine whether the BVP subtypes identified in this study have distinct prognoses or responses to treatment. Additionally, further research is warranted to explore the underlying mechanisms of these subtypes, including potential genetic or molecular factors that may predispose individuals to specific patterns of vestibular dysfunction. Such research could ultimately lead to more personalized approaches to diagnosing and managing BVP. Although we considered a bilateral horizontal aVOR gain pathological if <0.6, expanding the cutoff of the dysfunctional aVOR gain should be considered, according to the normative data we collected at the MSA ENT Academy Center Clinic in Cassino (FR), Italy. Indeed, the aVOR gain cutoff at 0.76 has a 100% sensitivity (69–100%) and 100% specificity (74–100%) in identifying the hypofunctional side [16,17].

We acknowledge that this study has some limitations that should be noted. First, unlike cVEMPs, there is no international consensus on ocular VEMPs in the literature. Second, our study did not include true negatives, thereby limiting the possibilities for analysis.

## 5. Conclusions

Our results confirm that several forms of BVH exist for which the application of the Barany criteria would not have allowed classification. These new variants of BVH must be considered. These bilateral lesions can also shed new light on the etiology and possible evolution of this nosological entity. It is therefore hoped that these criteria will be revisited as soon as possible, also in light of the instrumental resources available to clinicians today (vHIT and VEMPs).

## Figures and Tables

**Table 1:** Sample characteristics.

Female (*n*, %)	37 (47%)
Age (years ± SD)	61.40 ± 12.99
DHI (median, IQR)	67 (6)

DHI: Dizziness Handicap Inventory.

**Table 2:** Participants diagnosed with BPV, according to different criteria.

	BPV (*n*, %)
Barany criteria	36 (46)
Comprehensive model	78 (100)
Six-canals vHIT	59 (75)
oVEMPs dysfunction	52 (66)
cVEMPs dysfunction	41 (52)

BPV: bilateral vestibulopathy. The comprehensive model includes cVEMPs, oVEMPs, and six-canals vHIT.

**Table 3:** Multimodel diagnostic accuracy.

Index Test	Sensitivity	Specificity	LR+	LR−	ROC Area	95% CI
Six-canals vHIT	100%	45.24%	1.82	0.00	0.72	[0.65, 0.80]
oVEMPs	63.89%	30.95%	0.92	1.16	0.47	[0.36, 0.58]
cVEMPs	52.78%	47.62%	1.00	0.99	0.50	[0.38, 0.61]

**Table 4:** Barany criteria sensibility against the comprehensive model.

Index Test	Sensitivity	Specificity	LR+	LR−	ROC Area	95% CI
Barany criteria	46%	NC	NC	NC	NC	NC

NC: not calculable.

## Data Availability

Data are available under reasonable request to the corresponding author.
